# *SLC2A9* Genotype Is Associated with *SLC2A9* Gene Expression and Urinary Uric Acid Concentration

**DOI:** 10.1371/journal.pone.0128593

**Published:** 2015-07-13

**Authors:** Erin B. Ware, Ellen Riehle, Jennifer A. Smith, Wei Zhao, Stephen T. Turner, Sharon L. R. Kardia, John C. Lieske

**Affiliations:** 1 Department of Epidemiology, School of Public Health, University of Michigan, Ann Arbor, MI, United States of America; 2 Institute for Social Research, University of Michigan, Ann Arbor, MI, United States of America; 3 Division of Nephrology and Hypertension, Mayo Clinic, Rochester, MN, United States of America; 4 Department of Laboratory Medicine and Pathology, Mayo Clinic, Rochester, MN, United States of America; Case Western Reserve University, UNITED STATES

## Abstract

**Objectives:**

*SLC2A9* gene variants have been associated with urinary uric acid (UA) concentration, but little is known about the functional mechanism linking these gene variants with UA. *SLC2A9* encodes a UA transporter present in the proximal tubule of the kidney, and gene expression levels of *SLC2A9* and other genes in the uricosuric pathway (*ABCG2*, *SLC17A1*, *SLC17A3*, *and SLC22A12) * could potentially mediate the relationship between *SLC2A9* gene variants and urinary UA excretion.

**Methods:**

The association between urinary UA concentrations and single nucleotide polymorphisms (SNPs) within the *SLC2A9* gene region, expression levels of genes in the uricosuric pathway, and dietary protein intake were analyzed for a sample of non-Hispanic white participants from the Genetic Epidemiology Network of Arteriopathy (GENOA) cohort. The *SLC2A9 * SNP most significantly associated with urinary UA concentration was then tested for associations with gene expression levels from uric acid absorption/secretion associated genes. Models including interactions between dietary protein (total, animal, and vegetable) and genetic factors were also assessed.

**Results:**

The most significant *SLC2A9 * SNP associated with urinary UA (rs12509955, corrected p = 0.001) was also associated with *SLC2A9* gene expression levels (corrected p = 0.0084); however, *SLC2A9* gene expression levels were not significantly associated with urinary UA concentrations (p = 0.509). The interactions between rs12509955 and total dietary protein, and *SLC2A9 * gene-level gene expression and dietary vegetable protein on the outcome of urinary UA were marginally significant (p = 0.11 and p = 0.07, respectively). Gene expression level of one *SLC2A9* transcript had a significant interaction with dietary animal protein (*SLC2A9-001* ENST00000506583, p = 0.01) and a marginally significant interaction with total dietary protein (p = 0.07) on urinary UA.

**Conclusion:**

Our results illustrate that SNPs in the *SLC2A9* gene influence *SLC2A9* gene expression as well as urinary UA excretion. Evidence is also suggestive that gene-by-diet interactions may disproportionately increase urinary UA in genetically susceptible individuals that consume higher amounts of protein.

## Introduction

Nephrolithiasis (NL), or kidney stone formation, represents a substantial public health burden affecting approximately 1 in 11 Americans [1–3]. Specific diet patterns–particularly protein consumption–and familial history are both known risk factors for NL [4–11]. Most stones in the general population (70–80%) contain a majority of calcium oxalate (CaOx), while approximately 5–10% contain uric acid (UA) or a combination of UA and CaOx [3, 12, 13]. Hyperuricosuria is a risk factor for not only UA stone formation, but can also induce salting out of CaOx from solution, promoting CaOx stone formation [14–18]. Thus, factors influencing urinary UA excretion are likely important modifiers of kidney stone risk [11, 19, 20].

The concentration of UA in urine is determined by a combination of renal filtration followed by reabsorption and secretion in the nephron. *SLC2A9* encodes a key UA transporter expressed as two isoforms in the apical and basolateral membranes of the renal proximal tubule. This transporter also mediates uptake of UA from the blood into the liver [21–25]. Genetic variation in this gene has been associated with both mild and severe UA phenotypes, as well as NL [26–29]. Previous genome-wide association studies have identified single nucleotide polymorphisms (SNPs) within *SLC2A9* that are associated with serum and urinary UA concentration [30–36]. Several other genes have been found to be involved in excretion of UA in the urine as well as reabsorption within the kidney, though to a lesser extent than *SLC2A9*, and include *SLC22A12*, *SLC17A1*, *SLC17A3*, and *ABCG2* [37–40]. In addition, diet also plays a key role in UA homeostasis, and several studies have shown that higher protein (and purine) intake is associated with higher urinary UA concentration [41–44].

To better understand the contributions of genetic and dietary factors in urinary UA excretion, we examined the influence of *SLC2A9* gene variation on urinary UA, gene expression from uric acid absorption/secretion associated genes, and potential interactions with dietary protein. Since our gene expression measures are taken from transformed β lymphocytes, they are best thought of as a cumulative measure of the many genetic variations in each gene that are influencing its expression. Specifically, we identified the SNP within the *SLC2A9* gene region (rs12509955) that was most significantly associated with urinary UA excretion using a linear mixed model (LMM), and then examined its association with *SLC2A9*, *ABCG2*, *SLC17A1*, *SLC17A3*, and *SLC22A12* gene expression levels. We further investigated gene-by-dietary protein interaction effects on urinary UA to determine whether rs12509955 genotype or gene expression from uric acid absorption/secretion associated genes interacts with dietary protein intake, since purine metabolism is a known modifier of UA phenotypes.

## Methods

### Study Population

This study included non-Hispanic whites from Rochester, Minnesota that were enrolled in the Genetic Epidemiology Network of Arteriopathy (GENOA) study (Phase I: 1995–2000, n = 1,583; Phase II: 2000–2005, n = 1,241). GENOA is a multicenter, community-based study of hypertensive sibships initially collected to identify genes that influence blood pressure and target organ damage due to hypertension. The Genetic Determinants of Urinary Lithogenicity (GDUL) study (2008–2012) is an ancillary study of the Phase III GENOA Genetics of Chronic Kidney Disease Study (R01 DK073537), undertaken to investigate predictors of urinary supersaturation and risk of kidney stone diseases among participants without end-stage renal failure (Stage 5 Chronic Kidney Disease). GENOA participants were invited to join the GDUL study which consisted of a study visit; one (or preferably two or three) 24-hour urine collections for determination of quantitative urinary lithogenic factors, and a food frequency questionnaire (FFQ) (Viocare Technologies, Princeton, NJ, USA). Age, sex, and body mass index (BMI) were ascertained at the time of the GDUL exam. Of the 811 participants enrolled in GDUL, two were excluded from the initial SNP discovery analysis because their urinary UA concentration measure was >4 standard deviations from the mean. A subset who had gene expression data (n = 541 participants in 318 sibships) were used for all other association studies. A total of 424 of these participants also had animal and total dietary protein information (393 had vegetable protein) from the food frequency questionnaire. While urinary uric acid is the primary outcome of interest, blood serum uric acid was available from Phase II of the GENOA study, conducted approximately five years prior to the GDUL study. Serum uric acid was investigated to evaluate whether the effects of SNP variation and gene expression on urinary uric acid were consistent for serum uric acid. No participants were excluded for missing serum uric acid, as this was a supplemental analysis. All serum uric acid analyses can be found in supplemental material.

### Ethics Statement

This study protocol was approved by the Institutional Review Board at the Mayo Clinic under “Genetic Determinants of Urine Lithogenicity–Working Protocol” 08–006238.

### Urine Collection

After the initial visit, subjects completed one, two, or three 24-hour urine collections. Urine was collected with toluene as a preservative. Measurements were averaged for subjects with more than one 24-hour urine collection sample to account for the day-to-day variability in urinary composition. Twenty four-hour urine uric acid (mg/day) and sodium (mmol) concentrations were measured in the Mayo Clinic Renal Testing Laboratory. For additional details, see Lieske, et al. 2014 [45].

### Food frequency

Participants completed a food frequency questionnaire (Viocare Technologies, Princeton, NJ, USA) at a study visit. The Women's Health Initiative FFQ was adapted by Viocare in an electronic computer-administered format, and is similar to the Willett or Harvard Food Frequency Questionnaire. It assesses food intake over the last 3 months and employs the Minnesota NDS nutritional analysis database for nutrient analyses. The FFQ was validated and compares well with nutrient intake against 4-day food records and 24-hr dietary recalls [46]. The FFQ has validity for variables of interest to this study, when compared to a 4-day diet diary, namely for protein intake (r = 0.41) and sodium intake (r = 0.31). Dietary protein was assessed as total dietary protein, and also subdivided into animal or vegetable protein sources.

### Genotyping and Imputation

All GENOA participants were genotyped on the Affymetrix Genome-Wide Human SNP Array 6.0. A small portion of the GENOA stored blood samples contained poor quality DNA, and some of these were successfully genotyped using the Illumina Human 1M-Duo BeadChip. Since the two platforms used for genotyping contain only a small number of overlapping SNPs (~200,000), association analyses were performed using only imputed data. Prior to imputation, SNPs and samples with a call rate less than 95% were excluded. Imputation was performed using a single-step approach implemented in MACH v1.0.16 using the CEU reference panel of HapMap2 (release 22). The *SLC2A9* gene region of interest (including all SNPs within the gene, plus 200kb upstream and downstream) included a total of 880 SNPs. For analyses including the rs12509955 SNP, participants were categorized into imputed genotype (*i*) calls: (*i* <0.5 = 0; 0.5< *i* <1.5 = 1; *i*>1.5 = 2).

### Gene Expression Assessment and Quality Control

Blood samples for beta-lymphocyte extraction were collected from a subset of participants during GENOA Phase I and Phase II. RNA samples were extracted using standard protocols. RNA quality was assessed using the Agilent 2100 Bioanalyzer (Agilent Technologies Inc., Foster City, CA) and quantified by spectrophotometry using the Nanodrop ND-1000 (Nanodrop Inc., Wilmington, DE).

Array quality control was performed at the transcript level with the Affymetrix Expression Console (v 1.1) using core-level probe sets. All array images passed visual inspection. Hybridization controls were all detectable by signal increases that followed concentration. Signal intensity plots were examined for raw and processed data to identify outliers. Raw intensity data were processed using the Affymetrix Power Tool software. Probe summarization and set normalization were performed using Robust Multi-array Analysis (RMA), including background correction, quantile normalization, log_2_-transformation, and probe set summarization. Probe sets which are known to cross-hybridize and those with undetectable expression were also excluded. Gene-level expression was assessed by averaging all core probe sets for that gene. Transformed beta-lymphocyte gene expression measurements were adjusted for age, sex, and batch prior to analysis.

### Statistical Methods

Data management and statistical analyses were conducted in SAS version 9.3 [47]. Urinary phenotypes had approximately normal distributions; thus, no variable transformations were applied.

The sample of 809 GENOA participants with genotype and urinary UA excretion data was used to identify the SNP with the most significant p-value for association between SNP dosage (as an additive effect) and urinary UA. Fixed effects linear mixed modeling (LMM) was used to account for familial correlations within sibships. Age, sex, body mass index (BMI), and urinary sodium were included in the model because they significantly predict urinary UA in bivariate analyses (Table 1) or have been included as a covariate in other genetic models of urinary UA [32]. In order to investigate whether there were multiple independent signals within the *SLC2A9* region, we also tested for association between each SNP in the region and urinary UA, conditioned on the SNP mostly significantly associated with UA.

**Table 1:** Characteristics of study sample and bivariate associations with Urinary UA excretion and gene expression levels from uric acid absorption/secretion associated genes, n = 541.

		Urinary UA		Serum UA		*SLC2A9* Gene Expression	
	Mean (sd) / n (%)	B	P	B	P	B	P
Age (yrs)	65.5 (9.3)	-1.27	0.09	0.04	< .0001*	0.08	0.06
Male	215 (39.7%)	132.65	< .0001*	1.18	< .0001*	-0.01	0.78
BMI (kg/m^2^)	30.4 (5.6)	5.50	< .0001*	0.09	< .0001*	0.02	0.71
Urinary sodium (mmol)	138.6 (56.9)	1.76	< .0001*	0.01	< .0001*	0.00	0.93
Urinary UA (mg/day)	435.3 (161.7)			0.00	0.002*	0.03	0.51
Serum uric acid (mg/dL)	6.0 (1.6)	11.88	0.01*			0.05	0.28
**Dietary protein intake**							
Vegetable (g) (n = 393)	27.8 (13.3)	0.79	0.18	-0.002	0.74	0.03	0.59
Total (g) (n = 424)	80.5 (33.9)	0.82	<0.001*	0.002	0.27	-0.05	0.36
Animal (g) (n = 424)	52.6 (25.5)	1.25	<0.001*	0.004	0.13	-0.07	0.15
**Gene expression**							
*ABCG2*	4.6 (0.2)	-13.42	0.68	-0.11	0.72	0.32	< .0001*
*SLC17A1*	5.0 (0.2)	-31.93	0.34	0.11	0.72	0.40	< .0001*
*SLC17A3*	3.6 (0.3)	6.59	0.77	0.00	0.99	0.25	< .0001*
*SLC22A12*	6.7 (0.3)	-39.22	0.16	0.17	0.52	0.20	< .0001*
*SLC2A9*	5.4 (0.3)	17.95	0.51	0.27	0.30		
*SLC2A9-001* (n = 515)	5.7 (1.0)	1.72	0.82	0.02	0.74	0.01	0.58
*SLC2A9-002*	6.1 (1.0)	-3.85	0.59	-0.04	0.58	0.06	< .0001*
** Genotype**							
rs12509955		33.32	0.01*	-0.47	<0.001*	0.07	<0.001*
0	367 (67.8%)						
1	153 (28.3%)						
2	21 (3.9%)						

*statistically significant association at α = 0.05.

UA = uric acid; BMI = body mass index; Bivariate association results were obtained from linear mixed models accounting for sibship (n = 541). ABCG2 ENSG00000118777, SLC17A1 ENSG00000124568, SLC17A3 ENSG00000124564, SLC22A12 ENSG00000197891, SLC2A9 ENSG00000109667, SLC2A9-001 ENST00000506583, SLC2A9-201 ENST00000309065, SLC2A9-002 ENST00000264784.

β coefficients for association with gene expression were standardized.

In a subsample of 541 GDUL participants with gene expression data, LMM was used to assess whether there was an association between the rs12509955 genotype and gene expression levels from uric acid absorption/secretion associated genes. To further evaluate whether a genetic effect on gene expression levels had a corresponding influence on urinary UA, we used LMMs to test the association between gene expression and urinary UA excretion, adjusting for BMI and urinary sodium. LocusZoom plots were used to visualize results of the SNP associations with UA and gene expression [48].

To assess gene-by-diet interactions on urinary UA on rs12509955, LMMs were adjusted for age, sex, BMI, and urinary sodium, and included terms for SNP, dietary protein, and the interaction between dietary protein and SNP. Similarly, we assessed the potential interaction between each dietary protein measure and gene expression levels from uric acid absorption/secretion associated genes on urinary UA excretion. Interaction term β coefficients were considered to be statistically significant at an alpha level of 0.05.

## Results

### Descriptive Statistics

Table 1 provides descriptive statistics regarding demographic, anthropometric, dietary and genetic risk factors for the study sample and their regression relationships to urinary UA excretion and *SLC2A9* gene expression. In our sample, as expected, males had significantly higher urinary UA excretion (β = 132.6, p-value < 0.0001) compared to females. BMI and urinary sodium excretion were positively associated with urinary UA excretion (β = 5.5, p-value < 0.0001; β = 1.8, p-value < 0.0001). Total dietary protein and dietary animal protein were positively associated with urinary UA excretion (β = 0.8, p-value = 0.0003; β = 0.7, p-value = 0.004, respectively). Participants who were in the larger SNP discovery analysis sample (n = 809) but lacked gene expression data (n = 268) were not significantly different with regard to urinary UA excretion than the 541 participants with gene expression data (p-value = 0.10). Table 1 also displays the distribution of rs12509955 genotypes in our study sample (copies of the coded, minor allele (T): 0 = 67.8%; 1 = 28.3%; 2 = 3.9%) and their corresponding mean urinary UA excretion levels. The rs12509955 genotype was significantly associated with urinary UA (β = 33.32, p-value = 0.01), serum UA (β = -0.47, p-value <0.001), and *SLC2A9* gene expression (β = 0.07, p-value <0.001). Correlations for gene-level gene expression levels for the five investigated genes were moderate and significant (all p-values <0.0001) and ranged from r = -0.61 (*SLC2A9-001* and *SLC2A9-002*) to r = 0.55 (*SLC17A1* and *SLC17A3*). A correlation table is provided in the supplementary material (S1 Table).

### SLC2A9 SNP association with urinary UA excretion

**Fig 1:**
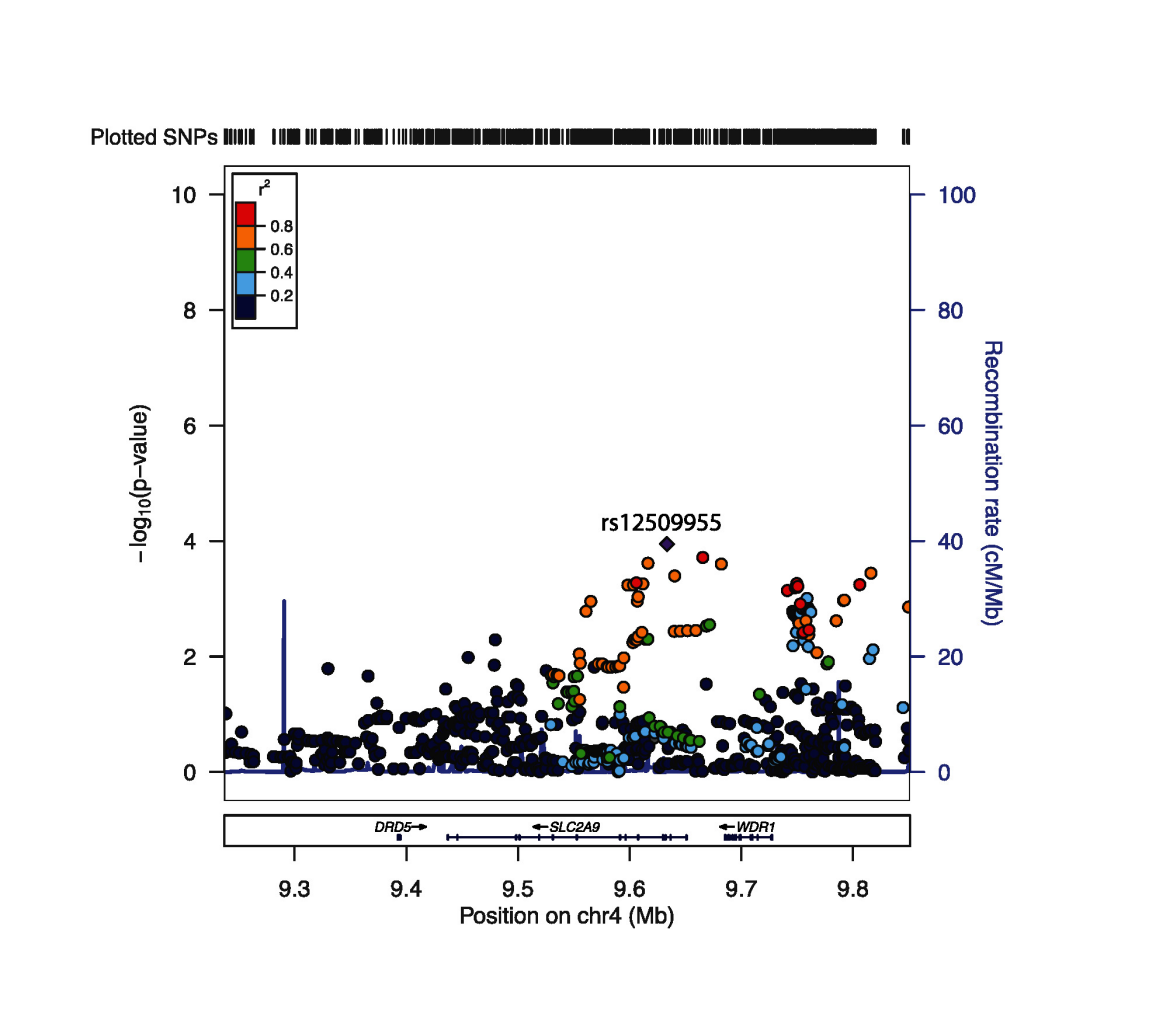
Urinary UA association results for 880 SNPs in the *SLC2A9* gene region. Left Y-axis:–log_10_(p-value) from association between SNPs and urinary UA, adjusted for age, sex, BMI, and urinary sodium, and accounting for sibship; Right Y-axis: SNP recombination rate based on HapMap hg18 CEU; X-axis: chromosomal location and gene regions; r^2^ color code: degree of linkage disequilibrium with index (most strongly associated) SNP, rs12509955 (purple diamond).

Of the 880 SNPs tested for an association with urinary UA (adjusted for sibships, age, sex, BMI, and urinary sodium in the LMM), the most significant SNP was rs12509955 (intron 2, coded allele = T, β = 40.0, corrected p-value = 0.001). P-values were corrected for the number of independent test in the *SLC2A9* gene region using principal components to account for correlated SNPs. After running a principal component analysis, 28 principal components were required to explain 95% of the variation in genotype and thus p-values were corrected by a factor of 28 [49]. As seen in Fig 1, the SCL2A9 gene region has a strong linkage disequilibrium pattern that extends across the *SLC2A9* gene, as well as the intergenic regions upstream. There are many SNPs that are strongly associated with urinary UA (S2 Table); however, after conditioning upon the most significant SNP, rs12509955, no additional SNPs in the region were significantly associated with urinary UA after multiple testing correction (S3 Table). We performed this analysis to detect any alternate independent predictive gene variants in the region in the context of a strong linkage disequilibrium pattern. Thus, for subsequent analyses, rs12509955 was selected to represent genetic variation in the *SLC2A9* region associated with urinary UA excretion. Since a subsample of 541 participants with gene expression data was used for the remainder of the analysis, we repeated the associations between SNPs and UA in this subsample and again found that rs12509955 was the most statistically significant (β = 40.1, corrected p-value = 0.001). Equivalent analyses for serum uric acid can be found in supplemental material (S4 Table and S5 Table, S1 Fig and S2 Fig).

### SLC2A9 SNP association with gene expression

**Fig 2:**
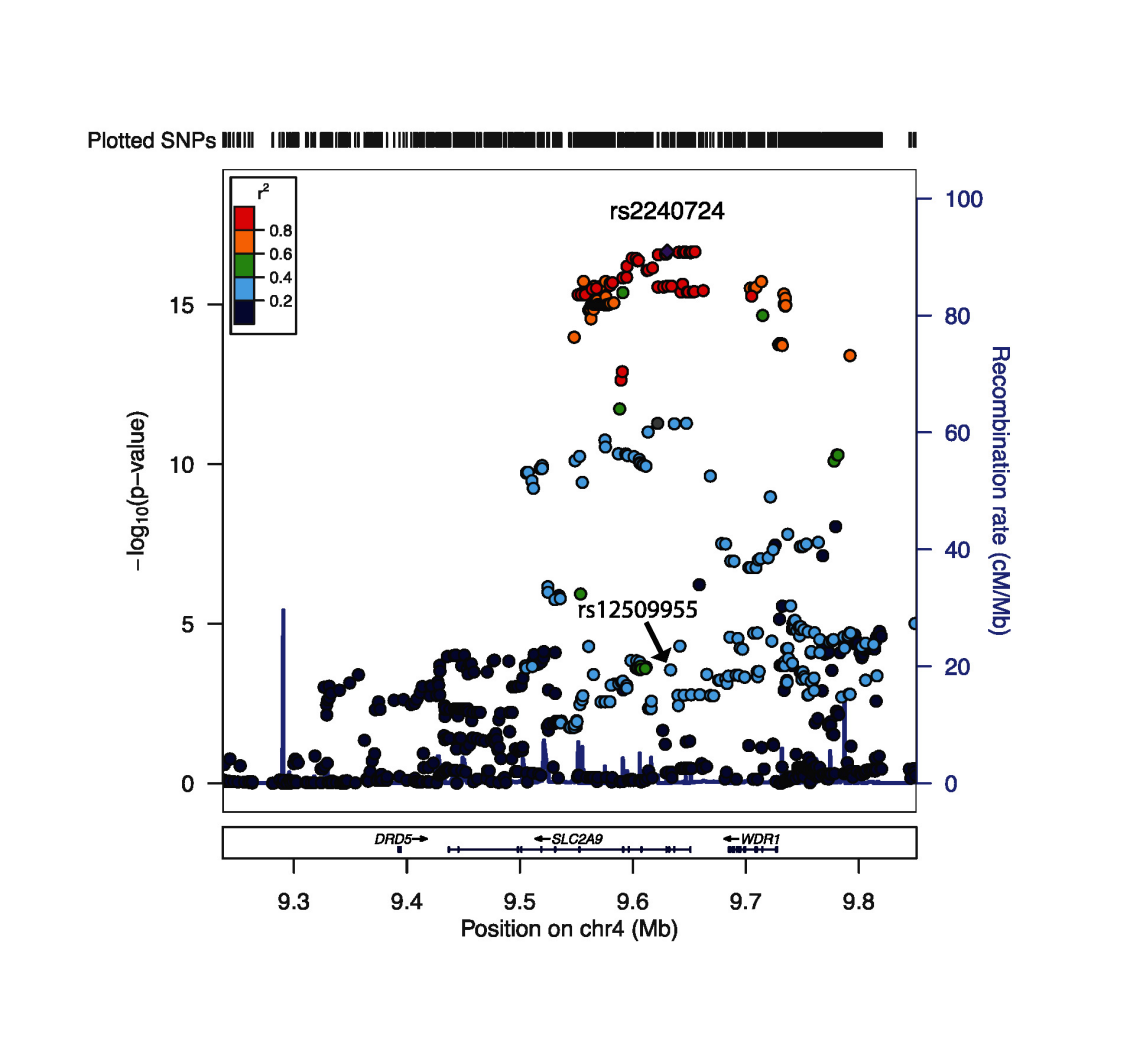
*SLC2A9* gene expression association results for 880 SNPs in the *SLC2A9* gene region. Left Y-axis:–log_10_(p-value) from association between SNPs and *SLC2A9* gene expression, accounting for sibship; Right Y-axis: SNP recombination rate based on HapMap hg18 CEU; X-axis: chromosomal location and gene regions; r^2^ color code: degree of linkage disequilibrium with index (most strongly associated) SNP, rs2240724 (purple diamond).

Next, 880 SNPs within the *SLC2A9* region were tested for association with *SLC2A9* gene expression. Of the 880 SNPs tested, 429 SNPs within the region were significantly associated with *SLC2A9* gene expression (corrected p-value < 0.05; Fig 2), with the most significant being rs2240724 (β = -0.08, corrected p-value = 6.10x10^-16^). As displayed in Table 1, a significant association between rs12509955 genotype and standardized *SLC2A9* gene-level gene expression was observed (β = 0.16, corrected p-value = 0.008). rs12509955 was not in linkage disequilibrium with rs2240724, and thus had an independent, more modest influence on gene expression than rs2240704 and the many other SNPs in the region that were associated with *SLC2A9* expression (Fig 2). rs2240704 was not investigated further because this SNP was not significantly and independently associated with urinary UA excretion, our main outcome of interest, in a model conditioned upon rs12509955. Overall, the tight linkage disequilibrium pattern indicated that gene expression levels are representing variation in over 48% of SNPs.

No SNPs in the *SLC2A9* gene region were significantly associated with gene expression of the other four genes of interest after multiple testing correction (α = 0.001). The rs12509955 genotype was not associated with gene expression for *SLC22A12* (β = 0.04, p-value = 1), *SLC17A1* (β = 0.02, p-value = 1), *SLC17A3* (β = 0.05, p-value = 0.75), or *ABCG2* (β = 0.00, p-value = 1), corrected for multiple testing.

### Gene expression association with urinary UA

*SLC2A9* gene expression level, representing *SLC2A9* gene variation across subjects, was not significantly associated with urinary UA excretion in either a bivariate model (Table 1, β = 18.0, p-value = 0.51) or a model adjusted for BMI and urinary sodium concentration (β = 15.5, p-value = 0.47). In bivariate models, none of the gene expressions were significantly associated with urinary uric acid (*ABCG2* (β = -13.4, p-value = 0.68)*; SLC17A1* (β = -31.9, p-value = 0.34); *SLC17A3* (β = 6.59, p-value = 0.77); *SLC22A12* (β = -39.2, p-value = 0.16)), nor was any gene’s expression significantly associated with urinary uric acid after adjustment for BMI and urinary sodium (*ABCG2* (β = -47.3, p-value = 0.06)*; SLC17A1* (β = -39.2, p-value = 0.14); *SLC17A3* (β = -12.6, p-value = 0.48); *SLC22A12* (β = -40.7, p-value = 0.06)), though both *ABCG2* and *SLC22A12* may be considered to have marginally significant effects. We did not adjust for age or sex in these models since gene expression had previously been adjusted for age, sex, and batch during quality control. Based on these marginally significant effects, indicating possible gene expression associations with urinary UA, we went on to investigate genetic interactions for all genes of interest (including transcript-based gene expression for the *SLC2A9* gene) with dietary protein. Equivalent analyses for serum uric acid can be found in supplemental material (S1 Text).

### Gene-by-dietary protein interactions

Models based upon urinary UA excretion and rs12509955-by-dietary protein interactions showed no significant interactions between dietary protein source and rs12509955 genotype, though the interaction between total protein and rs12509955 genotype was marginally significant (total protein: β = 0.50, p-value = 0.11; animal protein: β = 0.01, p-value = 0.98; vegetable protein β = 1.00, p-value = 0.27). Though not significant, those with two copies of the T allele had increased predicted urinary UA compared to those with one or those with no copies of the T allele at every level of dietary protein consumption for each type of dietary protein.

Interaction models (Table 2, serum UA S7 Table) examining the associations between gene expression and dietary protein, or the cumulative measure of variation in the gene and dietary protein, on urinary uric acid showed a marginally significant association between *SLC2A9* gene expression and dietary vegetable protein intake (β = 3.21, p-value = 0.07) and non-significant interactions with the other two protein sources (total protein: β = 1.00, p-value = 0.18; animal protein: β = 0.63, p-value = 0.52). No other genes showed significant or marginally significant interactions between gene expression and any of the dietary protein measures.

**Table 2:** Gene expression by dietary protein intake interaction associations for urinary uric acid.

	Urinary uric acid (mg/day)					
	Total protein		Animal protein		Vegetable protein	
	B	P	B	P	B	P
**ABCG2**	** **	** **	** **	** **	** **	** **
Protein	-1.78	0.71	-65.39	0.33	-11.95	0.34
Gene expression	-60.81	0.51	-2.78	0.59	-67.56	0.42
Protein x Gene expression	0.57	0.58	0.70	0.54	2.75	0.31
**SLC17A1**	** **	** **	** **	** **	** **	** **
Protein	0.59	0.92	-39.93	0.57	12.30	0.32
Gene expression	-26.19	0.79	-1.92	0.76	61.32	0.45
Protein x Gene expression	0.05	0.97	0.46	0.72	-2.27	0.35
**SLC17A3**	** **	** **	** **	** **	** **	** **
Protein	0.74	0.75	6.15	0.88	2.40	0.72
Gene expression	14.31	0.81	0.86	0.72	38.94	0.50
Protein x Gene expression	0.02	0.98	-0.13	0.84	-0.46	0.80
**SLC22A12**	** **	** **	** **	** **	** **	** **
Protein	8.90	0.11	48.47	0.38	2.10	0.88
Gene expression	55.42	0.46	10.06	0.10	-14.43	0.82
Protein x Gene expression	-1.20	0.14	-1.45	0.11	-0.18	0.93
**SLC2A9**	** **	** **	** **	** **	** **	** **
Protein	-5.17	0.20	-20.45	0.71	-17.33	0.07
Gene expression	-52.36	0.40	-4.76	0.38	-69.28	0.20
Protein x Gene expression	1.00	0.18	0.96	0.34	3.21	0.07
**SLC2A9-001**	** **	** **	** **	** **	** **	** **
Protein	-22.31	0.19	-25.51	0.08	1.67	0.92
Gene expression	-1.96	0.10	-3.41	0.02	-0.24	0.94
Protein x Gene expression	0.38	0.07	0.65	0.01*	0.06	0.92
**SLC2A9-002**	** **	** **	** **	** **	** **	** **
Protein	-5.88	0.70	2.70	0.83	-21.24	0.18
Gene expression	-0.01	1.00	0.99	0.47	-3.80	0.24
Protein x Gene expression	0.04	0.82	-0.11	0.64	0.67	0.21

*statistically significant association at α = 0.05.

ABCG2 ENSG00000118777, SLC17A1 ENSG00000124568, SLC17A3 ENSG00000124564, SLC22A12 ENSG00000197891, SLC2A9 ENSG00000109667, SLC2A9-001 ENST00000506583, SLC2A9-201 ENST00000309065, SLC2A9-002 ENST00000264784.

According to the literature, there are 13 splice variants in the *SLC2A9* gene region, four of which code for proteins [50]. In our data we observed three protein coding transcripts and two of them appeared at sufficient frequency (<5% missing data) to include in linear mixed models (*SLC2A9-001* ENST00000506583, bp = 1927, protein 511 aa, n = 515; *SLC2A9-002* ENST00000264784, bp = 1850, protein 540 aa, n = 541). These two transcripts are not significantly associated with urinary UA in bivariate models (*SLC2A9-001* β = 1.72, p-value = 0.82; *SLC2A9-002* β = -3.85, p-value = 0.59) or in models adjusted for BMI and urinary sodium (*SLC2A9-001* β = 3.95, p-value = 0.50; *SLC2A9-002* β = -2.24, p-value = 0.69). However, the *SLC2A9-001* gene expression transcript significantly interacts with dietary animal protein (β = 0.65, p-value = 0.01, S3 Fig) and has a marginally significant interaction with total dietary protein (β = 0.38, p-value = 0.07) in urinary UA models. The *SLC2A9-002* gene expression transcript significantly interacts with dietary total protein (β = -0.004, p-value = 0.04, S7 Table) and has marginally significant interactions with dietary animal and vegetable protein (β = -0.005, p-value = 0.07; β = -0.01, p-value = 0.06, respectively) in serum UA models (S7 Table).

## Discussion

SNP-level genetic variation within *SLC2A9* has previously been demonstrated to associate with urinary and serum UA phenotypes (30–36). Serum UA concentration and urinary UA excretion tend to be inversely related, since both are determined by the combination of UA reabsorption and secretion in the proximal tubule, and the *SLC2A9* urate transporter is a key mediator of this process. Indeed the top SNP we identified, rs1209955, was previously identified by Doring and colleagues as significantly associated with serum UA levels [22].

Our analysis demonstrated that the SNP most strongly associated with urinary UA excretion, rs12509955, also significantly associated with *SLC2A9* gene expression. This association between SNP-level variation within the *SLC2A9* region and *SLC2A9* gene expression has not previously been reported. The analysis also revealed other SNPs within the *SLC2A9* region that were even more significantly associated with *SLC2A9* gene expression than rs12509955.

One isoform of the SLCA29 transporter is expressed the apical membrane of the proximal tubule, while another is expressed in the basolateral membrane. The transporter exchanges UA for glucose and fructose resulting in net reabsorption of filtered UA. Genetic variation within *SLC2A9* has previously been associated with serum UA concentrations and fractional erection of UA into the urine. In the current study we confirm and extend these observations, and demonstrate that total urinary UA excretion is also influenced by genetic variation in *SLC2A9*. This observation implies that changes in UA generation occur when urinary fractional excretion of UA decreases [51]. Glut9, the protein product of *SLC2A9*, is required for hepatocyte UA uptake. In mice, live specific knockout of *SLC2A9* results in hyperuricemia and hyperuricosuria due to decreased degradation of UA by hepatic uricase. Humans lack uricase, but the current study suggests that genetic variability in *SLCA29* alters overall UA production, perhaps via feedback at the level of the liver on enzymatic pathways that result in its generation. It is also conceivable that changes in availability of fructose could alter UA generation [52].

We previously hypothesized that an association between *SLC2A9* genotype and UA phenotypes was mediated by *SLC2A9* gene expression. *SLC2A9* gene expression was not a significant predictor of urinary UA in our study. However, the gene expression data was obtained from transformed Beta-lymphocytes, and this cell type may not reflect gene expression levels present in the liver and kidneys, organs more directly responsible for UA metabolism and concentration. Nevertheless, the gene expression measured from transformed Beta-lymphocytes is thought to better reflect the proximal influences of genetic variation, since environmental modulators of gene expression are standardized by the uniform culture medium in which the cells are grown. It is quite possible that *SLC2A9* gene expression in kidney cells would have more variability, since both environmental and genetic variability would be contributing to gene expression. Specifically, environmental conditions, such as dietary protein consumption, could have profound effects on tissue level gene expression, but have limited impact on gene expression levels measured in transformed Beta-lymphocytes, except perhaps through epigenetic DNA modifications that persist post-transformation. Overall, gene expression measurements are more reflective of the cumulative effects of multiple variants in the gene effecting gene expression. This may explain why highly significant associations between SNP genotypes and gene-level gene expression were observed in the current study. However, numerous steps between gene/gene expression and urinary UA excretion substantially weaken the pathway of statistical association.

The current study identified one significant transcript-level gene expression-by-diet interaction and several marginally significant interactions influencing urinary UA excretion, and several significant gene expression-by-dietary protein interactions in the context of serum UA. Since so many SNPs influenced *SLC2A9* gene expression (Fig 2), it may be that gene expression values provide a better overall summary of the contribution common genetic variation in *SLC2A9* makes to UA excretion, and could explain why gene expression displays a greater interaction with diet than rs12509955 genotype. In the future, identifying means to capture the cumulative effects of many genetic variations in the *SLC2A9* region and their interactions with environmental factors such as dietary protein could facilitate efforts to assess the relative impact of genetic variability on serum and urinary UA levels.

In summary, the current study confirms that genetic variation within *SLC2A9* plays an important role in determining urinary UA excretion. The most significant SNP associated with urinary UA (rs12509955), and many other SNPs in the gene region, were associated with *SLC2A9* gene-level gene expression. Future investigations of mechanisms underlying associations between *SLC2A9* genetic variation and urinary UA should include efforts to understand the influence of diet on *SLC2A9* gene expression, urinary UA excretion, and NL risk.

## Supporting Information

S1 Fig Serum UA association results for 880 SNPs in the *SLC2A9* gene region. Left Y-axis:–log_10_(p-value) from association between SNPs and serum UA, adjusted for age, sex, BMI, and urinary sodium, and accounting for sibship; Right Y-axis: SNP recombination rate based on HapMap hg18 CEU; X-axis: chromosomal location and gene regions; r^2^ color code: degree of linkage disequilibrium with index (most strongly associated) SNP, rs11723439 (purple diamond).
(PDF)

S2 Fig Serum UA association results for 879 SNPs in the *SLC2A9* gene region, conditioned on rs11723439. Left Y-axis:–log_10_(p-value) from association between SNPs and serum UA, adjusted for age, sex, BMI, rs11723439 and urinary sodium, and accounting for sibship; Right Y-axis: SNP recombination rate based on HapMap hg18 CEU; X-axis: chromosomal location and gene regions; r^2^ color code: degree of linkage disequilibrium with index (most strongly associated) SNP, rs16894555 (purple diamond). Corrected p-value for rs16894555 is p = 0.044, indicating a second, independently SNP associated with serum uric acid in this sample.
(PDF)

S3 Fig *SLC2A9-001* gene expression transcript interaction plot with dietary animal protein on urinary uric acid. (PDF)

S1 Table Correlations between gene-level gene expression for uricosuric genes in the Genetic Epidemiology Network of Arteriopathy study. ABCG2 ENSG00000118777, SLC17A1 ENSG00000124568, SLC17A3 ENSG00000124564, SLC22A12 ENSG00000197891, SLC2A9 ENSG00000109667, SLC2A9-001 ENST00000506583, SLC2A9-201 ENST00000309065, SLC2A9-002 ENST00000264784.
(PDF)

S2 Table Forty *SLC2A9* SNPs with the lowest p-values in linear mixed models association with urinary uric acid in the Genetic Epidemiology Network of Arteriopathy study. *corrected for multiple testing. Linear mixed models adjusted for age, sex, BMI and urinary sodium and adjusted for sibships.
(PDF)

S3 Table Forty *SLC2A9* SNPs with the lowest p-values in linear mixed models, conditioned on rs12509955, association with urinary uric acid in the Genetic Epidemiology Network of Arteriopathy study. *corrected for multiple testing. Linear mixed models adjusted for age, sex, BMI and urinary sodium and adjusted for sibships.
(PDF)

S4 Table Forty *SLC2A9* SNPs with the lowest p-values in linear mixed models association with serum uric acid in the Genetic Epidemiology Network of Arteriopathy study. *corrected for multiple testing. Linear mixed models adjusted for age, sex, BMI and urinary sodium and adjusted for sibships.
(PDF)

S5 Table Forty *SLC2A9* SNPs with the lowest p-values in linear mixed models, conditioned on rs11723439, association with serum uric acid in the Genetic Epidemiology Network of Arteriopathy study. *corrected for multiple testing. Linear mixed models adjusted for age, sex, BMI and urinary sodium and adjusted for sibships.
(PDF)

S6 Table Forty *SLC2A9* SNPs with the lowest p-values in linear mixed models association with *SLC2A9* gene expression in the Genetic Epidemiology Network of Arteriopathy study. *corrected for multiple testing. Linear mixed models adjusted for BMI and urinary sodium and adjusted for sibships.
(PDF)

S7 Table Gene expression by dietary protein intake interaction associations for serum uric acid in the Genetic Epidemiology Network of Arteriopathy study. *statistically significant association at α = 0.05. ABCG2 ENSG00000118777, SLC17A1 ENSG00000124568, SLC17A3 ENSG00000124564, SLC22A12 ENSG00000197891, SLC2A9 ENSG00000109667, SLC2A9-001 ENST00000506583, SLC2A9-002 ENST00000264784.
(PDF)

S1 Text Gene expression association with serum UA. (PDF)
