# Supplementary figures and images for: SLC2A9 Genotype Is Associated with SLC2A9 Gene Expression and Urinary Uric Acid Concentration
Source: PLoS One. 2015 Jul 13;10(7):e0128593. doi: 10.1371/journal.pone.0128593 (PMC4500555; doi:10.1371/journal.pone.0128593)

Plotted SNPs

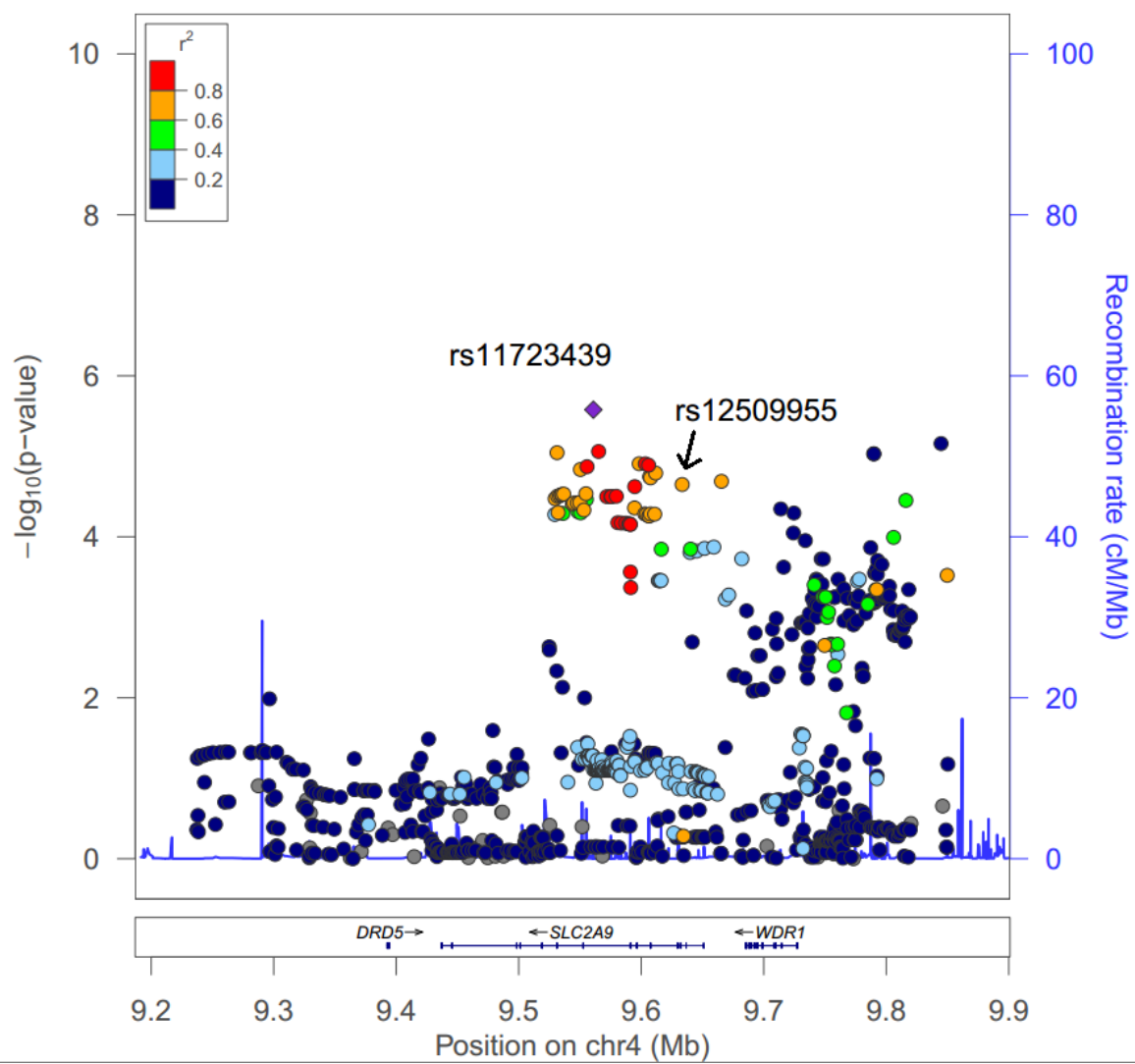

Supplement: S1 Fig — Left Y-axis:–log10(p-value) from association between SNPs and serum UA, adjusted for age, sex, BMI, and urinary sodium, and accounting for sibship; Right Y-axis: SNP recombination rate based on HapMap hg18 CEU; X-axis: chromosomal location and gene regions; r2 color code: degree of linkage disequilibrium with index (most strongly associated) SNP, rs11723439 (purple diamond). (PDF) [file pone.0128593.s001.pdf]

Plotted SNPs

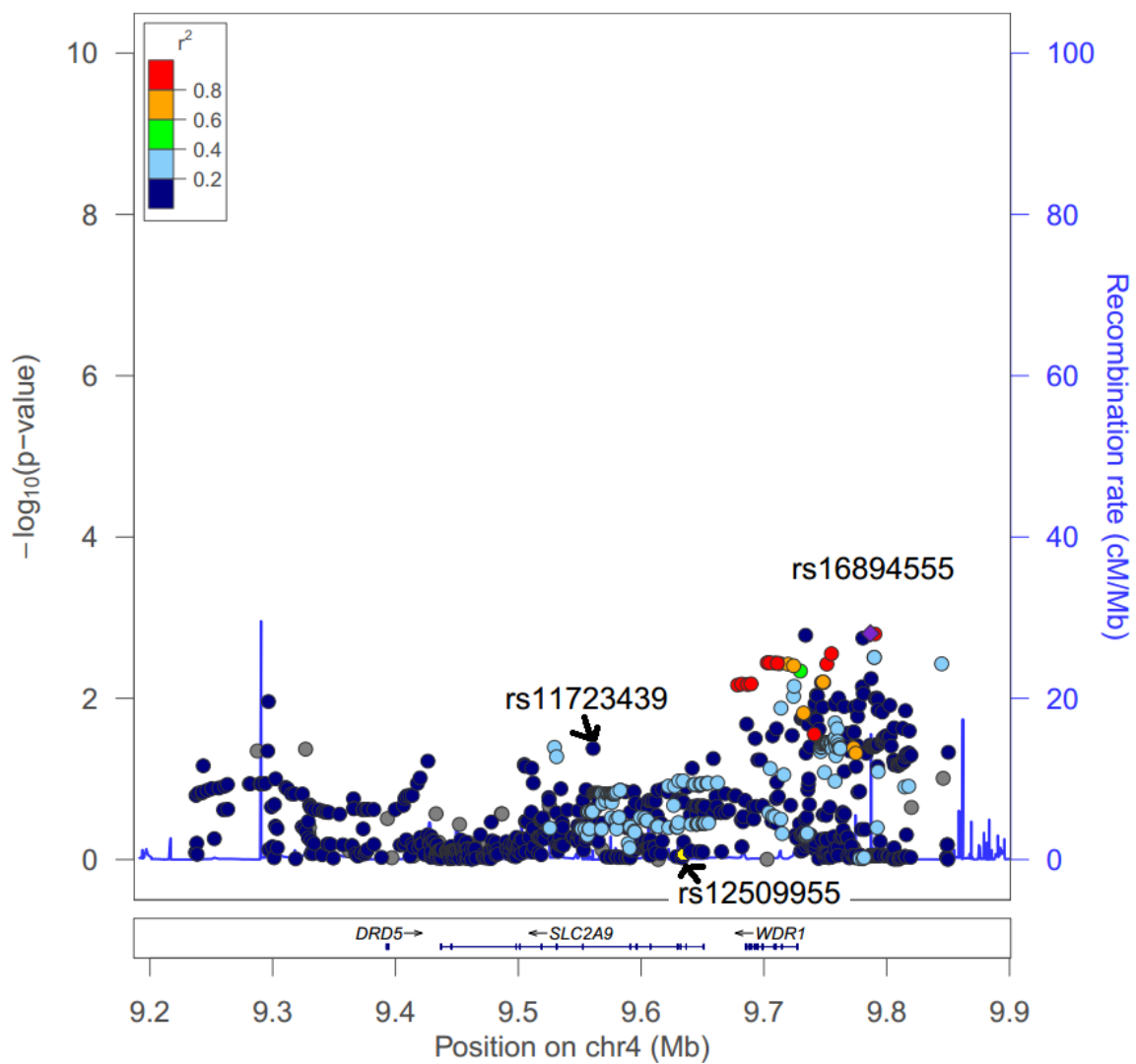

Supplement: S2 Fig — Left Y-axis:–log10(p-value) from association between SNPs and serum UA, adjusted for age, sex, BMI, rs11723439 and urinary sodium, and accounting for sibship; Right Y-axis: SNP recombination rate based on HapMap hg18 CEU; X-axis: chromosomal location and gene regions; r2 color code: degree of linkage disequilibrium with index (most strongly associated) SNP, rs16894555 (purple diamond). Corrected p-value for rs16894555 is p = 0.044, indicating a second, independently SNP associated with serum uric acid in this sample. (PDF) [file pone.0128593.s002.pdf]

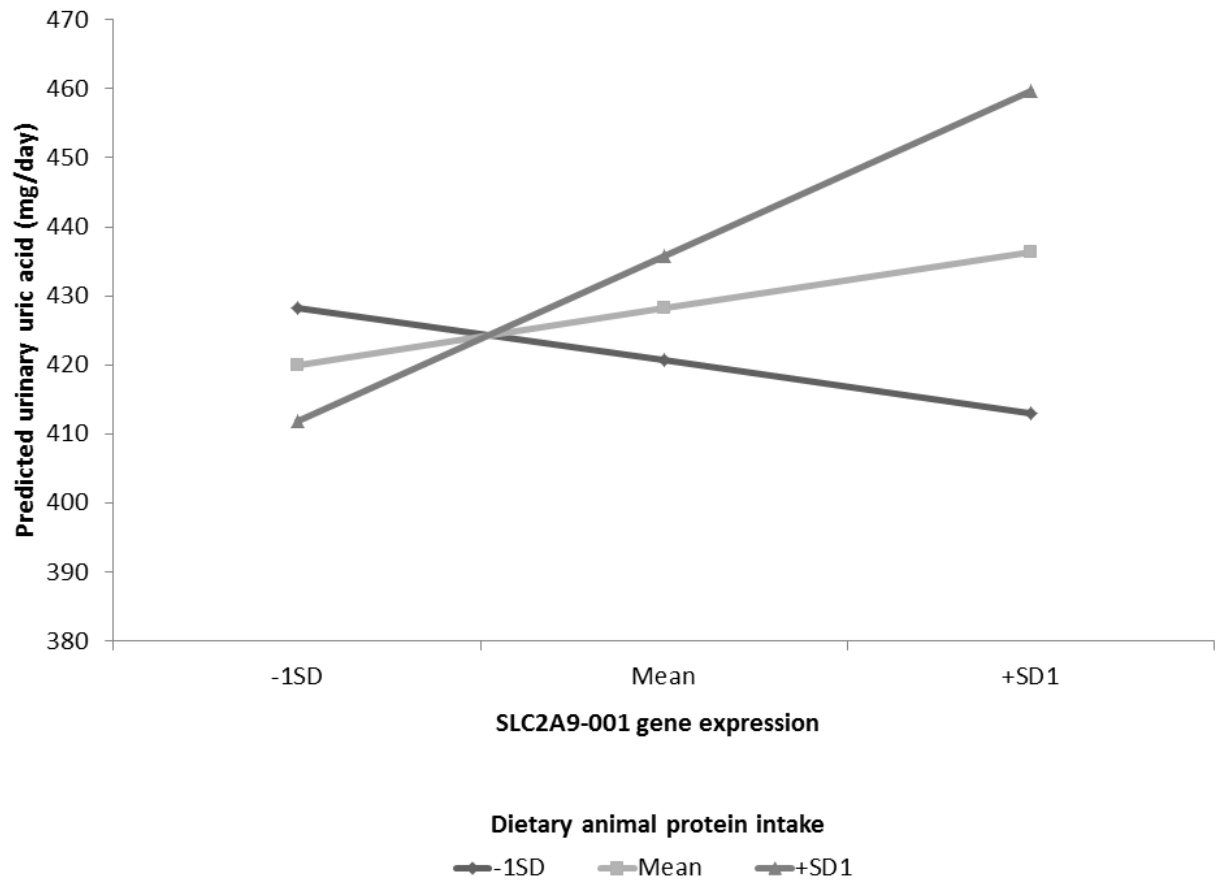

Supplement: S3 Fig — (PDF) [file pone.0128593.s003.pdf]
