# Supplementary material for: SLC2A9 Genotype Is Associated with SLC2A9 Gene Expression and Urinary Uric Acid Concentration
Source: PLoS One. 2015 Jul 13;10(7):e0128593. doi: 10.1371/journal.pone.0128593 (PMC4500555; doi:10.1371/journal.pone.0128593)
Supplement: S1 Table — ABCG2 ENSG00000118777, SLC17A1 ENSG00000124568, SLC17A3 ENSG00000124564, SLC22A12 ENSG00000197891, SLC2A9 ENSG00000109667, SLC2A9-001 ENST00000506583, SLC2A9-201 ENST00000309065, SLC2A9-002 ENST00000264784. (PDF) [file pone.0128593.s004.pdf]

	ABCG2	SLC17A1	SLC17A3	SLC22A12	SLC2A9	SLC2A9-001	SLC2A9-002
**ABCG2**	1	0.51	0.44	0.42	0.27	0.04	0.14
		<.0001	<.0001	<.0001	<.0001	0.41	0.00
**SLC17A1**		1	0.55	0.44	0.32	-0.06	0.20
			<.0001	<.0001	<.0001	0.17	<.0001
**SLC17A3**			1	0.38	0.30	-0.04	0.16
				<.0001	<.0001	0.40	0.00
**SLC22A12**				1	0.19	-0.03	0.10
					<.0001	0.57	0.02
**SLC2A9**					1	0.03	0.24
						0.57	<.0001
**SLC2A9-001**						1	-0.61
							<.0001
**SLC2A9-002**							1
