# Supplementary material for: SLC2A9 Genotype Is Associated with SLC2A9 Gene Expression and Urinary Uric Acid Concentration
Source: PLoS One. 2015 Jul 13;10(7):e0128593. doi: 10.1371/journal.pone.0128593 (PMC4500555; doi:10.1371/journal.pone.0128593)
Supplement: S2 Table — *corrected for multiple testing. Linear mixed models adjusted for age, sex, BMI and urinary sodium and adjusted for sibships. (PDF) [file pone.0128593.s005.pdf]

SNP	B	SE	T	P	P*
rs12509955	39.80	9.54	4.17	4.38E-05	0.001
rs9991278	-38.44	9.56	-4.02	8.04E-05	0.002
rs7671266	-38.36	9.60	-3.99	8.88E-05	0.002
rs4697700	36.13	9.06	3.99	9.07E-05	0.003
rs9291642	45.21	11.47	3.94	1.10E-04	0.003
rs7680126	-36.15	9.24	-3.91	1.23E-04	0.003
rs17389602	39.16	10.04	3.9	1.28E-04	0.004
rs887732	39.52	10.14	3.9	1.30E-04	0.004
rs13113918	37.29	9.58	3.89	1.31E-04	0.004
rs4697957	-39.50	10.14	-3.89	1.31E-04	0.004
rs4320137	43.92	11.28	3.89	1.32E-04	0.004
rs6449213	37.91	9.80	3.87	1.45E-04	0.004
rs4385059	37.90	9.80	3.87	1.46E-04	0.004
rs4475146	35.31	9.14	3.86	1.48E-04	0.004
rs12498742	-35.12	9.10	-3.86	1.50E-04	0.004
rs13111638	-37.79	9.80	-3.86	1.51E-04	0.004
rs7669607	-36.87	9.56	-3.86	1.52E-04	0.004
rs13145758	-35.77	9.40	-3.81	1.82E-04	0.005
rs13131257	-35.76	9.39	-3.81	1.83E-04	0.005
rs7376960	-34.92	9.19	-3.8	1.87E-04	0.005
rs11722229	-35.64	9.39	-3.8	1.91E-04	0.005
rs929575	-39.06	10.30	-3.79	1.93E-04	0.005
rs16868246	35.45	9.38	3.78	2.04E-04	0.006
rs1071988	-35.40	9.38	-3.77	2.07E-04	0.006
rs11942223	34.59	9.18	3.77	2.11E-04	0.006
rs6449173	34.59	9.18	3.77	2.11E-04	0.006
rs7442295	-34.59	9.18	-3.77	2.11E-04	0.006
rs9998811	34.59	9.18	3.77	2.11E-04	0.006
rs7439210	-35.26	9.37	-3.76	2.17E-04	0.006
rs6856396	42.86	11.44	3.75	2.29E-04	0.006
rs4481233	-35.85	9.83	-3.65	3.31E-04	0.009
rs734553	33.63	9.29	3.62	3.64E-04	0.010
rs6838021	-33.41	9.23	-3.62	3.66E-04	0.010
rs938555	33.42	9.25	3.61	3.77E-04	0.011
rs938554	33.41	9.27	3.61	3.86E-04	0.011
rs6832439	33.38	9.28	3.6	3.99E-04	0.011
rs938564	33.34	9.29	3.59	4.10E-04	0.011
rs4697913	-32.61	9.09	-3.59	4.12E-04	0.012
rs7660895	32.13	8.99	3.57	4.33E-04	0.012
