# Supplementary material for: SLC2A9 Genotype Is Associated with SLC2A9 Gene Expression and Urinary Uric Acid Concentration
Source: PLoS One. 2015 Jul 13;10(7):e0128593. doi: 10.1371/journal.pone.0128593 (PMC4500555; doi:10.1371/journal.pone.0128593)
Supplement: S3 Table — *corrected for multiple testing. Linear mixed models adjusted for age, sex, BMI and urinary sodium and adjusted for sibships. (PDF) [file pone.0128593.s006.pdf]

SNP	B	SE	T	P	P*
rs4461524	-23.32	9.14	-2.55	0.011	0.319
rs6449286	21.39	8.54	2.51	0.013	0.364
rs4467562	20.70	8.36	2.47	0.014	0.395
rs10001106	19.91	8.40	2.37	0.019	0.521
rs6816053	227.72	100.20	2.27	0.024	0.672
rs17450260	-19.36	8.93	-2.17	0.031	0.874
rs17450372	19.25	8.93	2.16	0.032	0.902
rs4697936	17.87	8.71	2.05	0.041	1.000
rs1009144	-17.70	8.99	-1.97	0.050	1.000
rs17450434	-17.67	8.99	-1.97	0.051	1.000
rs12513376	-17.10	8.76	-1.95	0.052	1.000
rs6851524	-17.88	9.19	-1.95	0.053	1.000
rs4619888	-17.68	9.14	-1.93	0.054	1.000
rs11929718	-15.87	8.28	-1.92	0.057	1.000
rs715260	-17.52	9.16	-1.91	0.057	1.000
rs17385294	16.92	8.99	1.88	0.061	1.000
rs12507586	16.75	8.92	1.88	0.062	1.000
rs6814556	16.53	8.87	1.86	0.064	1.000
rs11724641	-15.62	8.38	-1.86	0.064	1.000
rs3822236	-16.80	9.05	-1.86	0.065	1.000
rs12508413	16.52	8.91	1.85	0.065	1.000
rs6813334	16.49	8.90	1.85	0.065	1.000
rs4292329	16.65	9.15	1.82	0.070	1.000
rs7667775	-17.16	9.43	-1.82	0.070	1.000
rs11734209	-15.99	8.83	-1.81	0.072	1.000
rs4697926	15.67	8.66	1.81	0.072	1.000
rs747356	-15.20	8.43	-1.80	0.073	1.000
rs731069	-15.17	8.42	-1.80	0.073	1.000
rs731070	-15.16	8.42	-1.80	0.073	1.000
rs4697940	15.15	8.42	1.80	0.073	1.000
rs747357	-15.15	8.42	-1.80	0.073	1.000
rs881642	-16.26	9.05	-1.80	0.074	1.000
rs715979	-16.47	9.18	-1.79	0.074	1.000
rs12501597	-16.24	9.06	-1.79	0.074	1.000
rs6449289	-18.57	10.39	-1.79	0.075	1.000
rs7659717	15.33	8.66	1.77	0.078	1.000
rs4235356	-15.93	9.01	-1.77	0.078	1.000
rs9291645	-14.74	8.34	-1.77	0.079	1.000
rs4235355	-15.90	9.00	-1.77	0.079	1.000
