# Supplementary material for: SLC2A9 Genotype Is Associated with SLC2A9 Gene Expression and Urinary Uric Acid Concentration
Source: PLoS One. 2015 Jul 13;10(7):e0128593. doi: 10.1371/journal.pone.0128593 (PMC4500555; doi:10.1371/journal.pone.0128593)
Supplement: S4 Table — *corrected for multiple testing. Linear mixed models adjusted for age, sex, BMI and urinary sodium and adjusted for sibships. (PDF) [file pone.0128593.s007.pdf]

SNP	B	SE	T	P	P*
rs11723439	0.52	0.11	4.83	2.64E-06	7.39E-05
rs2080072	-0.39	0.08	-4.61	6.95E-06	1.95E-04
rs4481233	0.49	0.11	4.56	8.72E-06	2.44E-04
rs16890979	0.46	0.10	4.55	9.02E-06	2.52E-04
rs11724112	-0.39	0.08	-4.54	9.31E-06	2.61E-04
rs6811287	0.39	0.08	4.54	9.33E-06	2.61E-04
rs4385059	-0.48	0.11	-4.48	1.24E-05	3.46E-04
rs6449213	-0.48	0.11	-4.48	1.24E-05	3.47E-04
rs13111638	0.48	0.11	4.47	1.29E-05	3.61E-04
rs4475146	-0.45	0.10	-4.46	1.35E-05	3.77E-04
rs4697913	0.44	0.10	4.44	1.46E-05	4.08E-04
rs9991278	0.46	0.10	4.41	1.62E-05	4.53E-04
rs7669607	0.46	0.10	4.39	1.79E-05	5.01E-04
rs13113918	-0.46	0.10	-4.38	1.85E-05	5.18E-04
rs7671266	0.46	0.11	4.36	2.05E-05	5.73E-04
rs12509955	-0.45	0.10	-4.34	2.24E-05	6.27E-04
rs7680126	0.44	0.10	4.32	2.38E-05	6.67E-04
rs4697700	-0.43	0.10	-4.27	2.91E-05	8.16E-04
rs6838021	0.43	0.10	4.27	2.95E-05	8.25E-04
rs938555	-0.43	0.10	-4.27	2.98E-05	8.35E-04
rs938554	-0.43	0.10	-4.26	3.02E-05	8.45E-04
rs6832439	-0.43	0.10	-4.26	3.08E-05	8.62E-04
rs938564	-0.43	0.10	-4.25	3.13E-05	8.75E-04
rs7376960	0.43	0.10	4.25	3.14E-05	8.78E-04
rs11942223	-0.43	0.10	-4.25	3.17E-05	8.87E-04
rs6449173	-0.43	0.10	-4.25	3.17E-05	8.87E-04
rs9998811	-0.43	0.10	-4.25	3.17E-05	8.87E-04
rs7442295	0.43	0.10	4.25	3.17E-05	8.87E-04
rs874432	-0.43	0.10	-4.24	3.36E-05	9.41E-04
rs4697701	-0.41	0.10	-4.23	3.43E-05	9.61E-04
rs17389602	-0.47	0.11	-4.23	3.53E-05	9.89E-04
rs5028843	-0.41	0.10	-4.21	3.74E-05	1.05E-03
rs7670751	0.41	0.10	4.21	3.80E-05	1.06E-03
rs6855911	0.41	0.10	4.21	3.81E-05	1.07E-03
rs737267	0.41	0.10	4.20	3.91E-05	1.10E-03
rs7660895	-0.42	0.10	-4.17	4.38E-05	1.23E-03
rs717614	0.35	0.08	4.17	4.51E-05	1.26E-03
rs12498742	0.42	0.10	4.16	4.66E-05	1.30E-03
rs938558	0.39	0.09	4.15	4.87E-05	1.36E-03
