# Supplementary material for: SLC2A9 Genotype Is Associated with SLC2A9 Gene Expression and Urinary Uric Acid Concentration
Source: PLoS One. 2015 Jul 13;10(7):e0128593. doi: 10.1371/journal.pone.0128593 (PMC4500555; doi:10.1371/journal.pone.0128593)
Supplement: S5 Table — *corrected for multiple testing. Linear mixed models adjusted for age, sex, BMI and urinary sodium and adjusted for sibships. (PDF) [file pone.0128593.s008.pdf]

SNP	B	SE	T	P	P*
rs16894555	0.31	0.10	3.20	0.002	0.044
rs714814	-0.31	0.10	-3.20	0.002	0.045
rs4456954	0.36	0.11	3.19	0.002	0.046
rs7686718	0.29	0.09	3.16	0.002	0.050
rs10024447	0.27	0.09	3.02	0.003	0.078
rs11724112	-0.27	0.09	-2.99	0.003	0.087
rs6811287	0.27	0.09	2.99	0.003	0.087
rs3822239	0.28	0.09	2.94	0.004	0.101
rs3796822	0.28	0.09	2.94	0.004	0.101
rs2241482	-0.28	0.09	-2.94	0.004	0.102
rs4697917	0.28	0.09	2.94	0.004	0.103
rs12499240	0.28	0.09	2.93	0.004	0.104
rs2080072	-0.27	0.09	-2.93	0.004	0.105
rs16894053	-0.27	0.09	-2.93	0.004	0.105
rs4697922	0.28	0.09	2.93	0.004	0.106
rs4393994	-0.27	0.09	-2.91	0.004	0.111
rs12374320	0.27	0.09	2.86	0.005	0.129
rs17385872	0.26	0.09	2.79	0.006	0.160
rs12646146	0.26	0.10	2.76	0.006	0.176
rs6449300	-0.27	0.10	-2.76	0.006	0.178
rs12507725	0.27	0.10	2.76	0.006	0.178
rs2241469	0.26	0.10	2.74	0.007	0.185
rs7667452	0.26	0.10	2.74	0.007	0.186
rs3775935	-0.26	0.10	-2.74	0.007	0.189
rs3775938	0.26	0.10	2.73	0.007	0.192
rs12498927	-0.24	0.09	-2.72	0.007	0.198
rs874079	0.23	0.08	2.71	0.007	0.201
rs2080076	-0.22	0.08	-2.65	0.009	0.243
rs2098234	-0.22	0.08	-2.65	0.009	0.245
rs881641	0.26	0.10	2.63	0.009	0.255
rs1109472	0.26	0.10	2.62	0.009	0.261
rs17197769	-0.26	0.10	-2.62	0.009	0.261
rs4604059	0.23	0.09	2.62	0.009	0.265
rs887735	0.25	0.10	2.60	0.010	0.278
rs6827496	-0.26	0.10	-2.60	0.010	0.278
rs12500891	0.25	0.10	2.60	0.010	0.281
rs887729	-0.25	0.10	-2.59	0.010	0.289
rs7671092	-0.51	0.20	-2.57	0.011	0.307
rs6449289	-0.29	0.11	-2.54	0.012	0.326
