# Supplementary material for: SLC2A9 Genotype Is Associated with SLC2A9 Gene Expression and Urinary Uric Acid Concentration
Source: PLoS One. 2015 Jul 13;10(7):e0128593. doi: 10.1371/journal.pone.0128593 (PMC4500555; doi:10.1371/journal.pone.0128593)
Supplement: S6 Table — *corrected for multiple testing. Linear mixed models adjusted for BMI and urinary sodium and adjusted for sibships. (PDF) [file pone.0128593.s009.pdf]

SNP	B	SE	T	P	P*
rs2240724	-0.13	0.01	-9.18	3.28E-17	9.19E-16
rs10939669	0.13	0.01	9.18	3.29E-17	9.21E-16
rs13137343	-0.13	0.01	-9.17	3.38E-17	9.47E-16
rs11731110	-0.13	0.01	-9.17	3.4E-17	9.52E-16
rs10939665	-0.13	0.01	-9.17	3.4E-17	9.53E-16
rs11727199	0.13	0.01	9.17	3.41E-17	9.54E-16
rs3733585	0.13	0.01	9.17	3.41E-17	9.54E-16
rs13101785	-0.13	0.01	-9.17	3.43E-17	9.59E-16
rs11722930	-0.13	0.01	-9.17	3.44E-17	9.63E-16
rs12506455	-0.13	0.01	-9.17	3.45E-17	9.64E-16
rs12508991	0.13	0.01	9.17	3.46E-17	9.69E-16
rs7679916	0.13	0.01	9.17	3.53E-17	9.88E-16
rs2240721	-0.14	0.01	-9.16	3.78E-17	1.06E-15
rs2240720	0.14	0.01	9.16	3.82E-17	1.07E-15
rs13133766	0.13	0.01	9.15	3.89E-17	1.09E-15
rs3756236	-0.13	0.01	-9.15	4.04E-17	1.13E-15
rs17187075	-0.13	0.01	-9.11	5.23E-17	1.46E-15
rs7678012	-0.13	0.01	-9.11	5.3E-17	1.48E-15
rs3796842	0.13	0.01	9.08	6.17E-17	1.73E-15
rs12499857	-0.13	0.01	-9.08	6.35E-17	1.78E-15
rs17246501	0.13	0.01	9.03	8.83E-17	2.47E-15
rs4543113	-0.13	0.01	-9.02	9.39E-17	2.63E-15
rs17247314	0.13	0.01	8.99	1.19E-16	3.34E-15
rs4622999	0.13	0.01	8.98	1.23E-16	3.44E-15
rs717615	-0.13	0.01	-8.91	1.94E-16	5.44E-15
rs11723591	-0.13	0.01	-8.91	1.94E-16	5.45E-15
rs13115193	0.13	0.01	8.91	2E-16	5.61E-15
rs1122141	0.13	0.02	8.87	2.51E-16	7.03E-15
rs7663097	0.13	0.01	8.87	2.59E-16	7.24E-15
rs13103690	0.13	0.01	8.85	2.85E-16	7.98E-15
rs13103879	0.13	0.01	8.85	2.85E-16	7.99E-15
rs6449183	0.13	0.01	8.85	2.92E-16	8.17E-15
rs17245723	-0.13	0.01	-8.85	2.99E-16	8.36E-15
rs6449156	0.13	0.01	8.85	2.99E-16	8.37E-15
rs2241483	-0.13	0.01	-8.84	3.08E-16	8.62E-15
rs13146686	-0.13	0.01	-8.83	3.26E-16	9.13E-15
rs11726271	0.13	0.01	8.83	3.26E-16	9.14E-15
rs3822242	0.13	0.01	8.83	3.29E-16	9.21E-15
rs4292328	-0.13	0.01	-8.83	3.3E-16	9.25E-15
