# Supplementary material for: SLC2A9 Genotype Is Associated with SLC2A9 Gene Expression and Urinary Uric Acid Concentration
Source: PLoS One. 2015 Jul 13;10(7):e0128593. doi: 10.1371/journal.pone.0128593 (PMC4500555; doi:10.1371/journal.pone.0128593)
Supplement: S7 Table — *statistically significant association at α = 0.05. ABCG2 ENSG00000118777, SLC17A1 ENSG00000124568, SLC17A3 ENSG00000124564, SLC22A12 ENSG00000197891, SLC2A9 ENSG00000109667, SLC2A9-001 ENST00000506583, SLC2A9-002 ENST00000264784. (PDF) [file pone.0128593.s010.pdf]

	Serum uric acid (mg/dL)					
	**Total protein**		**Animal protein**		**Vegetable protein**	
	**B**	**P**	**B**	**P**	**B**	**P**
**ABCG2**						
Protein	0.28	0.74	0.28	0.72	0.25	0.74
Gene expression	0.02	0.69	0.03	0.65	0.01	0.90
Protein * Gene expression	0.00	0.67	-0.01	0.63	0.00	0.88
**SLC17A1**						
Protein	1.13	0.21	1.05	0.18	0.36	0.63
Gene expression	0.06	0.29	0.08	0.25	0.02	0.86
Protein * Gene expression	-0.01	0.28	-0.02	0.24	0.00	0.83
**SLC17A3**						
Protein	0.00	1.00	0.19	0.67	-0.55	0.30
Gene expression	0.00	0.89	0.01	0.73	-0.08	0.17
Protein * Gene expression	0.00	0.93	-0.003	0.69	0.02	0.19
**SLC22A12**						
Protein	-0.23	0.73	-0.02	0.98	-0.45	0.44
Gene expression	-0.04	0.49	-0.03	0.70	-0.15	0.21
Protein * Gene expression	0.01	0.50	0.004	0.72	0.02	0.22
**SLC2A9**						
Protein	-0.15	0.83	0.21	0.73	-0.69	0.28
Gene expression	-0.05	0.27	-0.04	0.53	-0.24	0.03
Protein * Gene expression	0.01	0.28	0.01	0.54	0.04	0.04*
**SLC2A9-001**						
Protein	-0.15	0.44	-0.12	0.48	-0.31	0.14
Gene expression	-0.01	0.29	-0.02	0.29	-0.06	0.12
Protein * Gene expression	0.00	0.30	0.00	0.30	0.01	0.14
**SLC2A9-002**						
Protein	0.34	0.05	0.24	0.10	0.38	0.04
Gene expression	0.02	0.05	0.03	0.09	0.07	0.08
Protein * Gene expression	-0.004	0.04*	-0.005	0.07	-0.01	0.06
