# Supplementary material for: SLC2A9 Genotype Is Associated with SLC2A9 Gene Expression and Urinary Uric Acid Concentration
Source: PLoS One. 2015 Jul 13;10(7):e0128593. doi: 10.1371/journal.pone.0128593 (PMC4500555; doi:10.1371/journal.pone.0128593)
Supplement: S1 Text — (PDF) [file pone.0128593.s011.pdf]

*SLC2A9* gene expression level was not significantly associated with serum UA excretion in either a bivariate model ($\beta = 0.27$, p-value = 0.30) or a model adjusted for BMI and urinary sodium concentration ($\beta = 0.28$, p-value = 0.28). In bivariate models, none of the gene expressions was significantly associated with urinary uric acid (*ABCG2* ($\beta$=-0.11, p-value=0.72)*; SLC17A1* ($\beta$=0.11, p-value=0.72);  *SLC17A3* ($\beta$=0.00, p-value=0.99); *SLC22A12* ($\beta$=0.17, p-value=0.52)), nor was any gene's expression significantly associated with urinary uric acid after adjustment for BMI and urinary sodium (*ABCG2* ($\beta$=-0.30, p-value=0.29)*; SLC17A1* ($\beta$=0.04, p-value=0.90);  *SLC17A3* ($\beta$=-0.04, p-value=0.84); *SLC22A12* ($\beta$=0.13, p-value=0.60)). We did not adjust for age or sex in these models since gene expression had previously been adjusted for age, sex, and batch during quality control.
